# Contractile Behavior of Mouse Aorta Depends on SERCA2 Isoform Distribution: Effects of Replacing SERCA2a by SERCA2b

**DOI:** 10.3389/fphys.2020.00282

**Published:** 2020-03-31

**Authors:** Paul Fransen, Jialin Chen, Peter Vangheluwe, Pieter-Jan Guns

**Affiliations:** ^1^Laboratory of Physiopharmacology, Faculty of Pharmaceutical Sciences and Faculty of Medicine and Health Sciences, University of Antwerp, Antwerp, Belgium; ^2^Laboratory of Cellular Transport Systems, KU Leuven, Leuven, Belgium

**Keywords:** SERCA isoforms, vascular smooth muscle, contraction, mouse aorta, intracellular calcium

## Abstract

The Sarco(endo)plasmic reticulum Ca^2+^ ATPase (SERCA) actively pumps Ca^2+^ into the sarco/endoplasmic reticulum, thereby regulating intracellular Ca^2+^ concentrations and associated physiological processes. Different SERCA isoforms have been described (SERCA1, 2, and 3) with SERCA2 playing a pivotal role in Ca^2+^ homeostasis in cardiovascular tissues. In the heart, SERCA2a is the dominant isoform and has been proposed as therapeutic target in patients with heart failure. In the vasculature, both SERCA2a and SERCA2b are expressed with SERCA2b being the predominant isoform. The physiological role of SERCA2a in the vasculature, however, remains incompletely understood. In the present study, we used gene-modified mice in which the alternative splicing of the SERCA2-encoding gene (*Atp2a2*), underlying the expression of SERCA2a, is prevented and SERCA2a is replaced by SERCA2b. The resulting SERCA2^b/b^ mice provide a unique opportunity to investigate the specific contribution of SERCA2a versus SERCA2b to vascular physiology. Aortic segments of SERCA2^b/b^ (SERCA2a-deficient) and SERCA2^a/b^ (control) mice were mounted in organ baths to evaluate vascular reactivity. SERCA2^b/b^ aortic rings displayed higher contractions induced by phenylephrine (1 μM). Surprisingly, the initial inositol-3-phosphate mediated phasic contraction showed a faster decay of force in SERCA2^b/b^ mice, while the subsequent tonic contraction was larger in SERCA2^b/b^ segments. Moreover, in the presence of the calcium channel blocker diltiazem (35 μM) SERCA2^b/b^ aortic rings showed higher contractions compared to SERCA2^a/b^, suggesting that SERCA2a (deficiency) modulates the activity of non-selective cation channels. Additionally, in endothelial cell (EC)-denuded aortic segments, the SERCA-inhibitor cyclopiazonic acid (CPA) caused markedly larger contractions in SERCA2^b/b^ mice, while the increases of cytosolic Ca^2+^ were similar in both strains. Hence, aortas of SERCA2^b/b^ mice appear to have a stronger coupling of intracellular Ca^2+^ to contraction, which may be in agreement with the reported difference in intracellular localization of SERCA2a versus SERCA2b. Finally, EC-mediated relaxation by acetylcholine and ATP was assessed. Concentration-response-curves for ATP showed a higher sensitivity of aortic segments of SERCA2^b/b^ mice, while no difference in potency between strains were observed for acetylcholine. In summary, despite the relative low expression of SERCA2a in the murine aorta, our results point toward a distinct role in vascular physiology.

## Introduction

Cytosolic calcium (Ca^2+^) mobilization is a key event controlling multiple physiological processes in the blood vessel wall. Whereas in endothelial cells (EC) Ca^2+^ activates NO-synthase (eNOS) to produce the relaxing factor nitric oxide (NO), Ca^2+^ mobilization in smooth muscle cells (SMC) controls contraction and relaxation (Berridge, 2008). In both cell types, raised activator Ca^2+^ has to be cleared from the cytoplasm upon subsequent inactivation. To ensure cytoplasmic Ca^2+^ clearance and, hence, EC and SMC inactivation, the plasmalemmal Ca^2+^ ATPases (PMCA), and the Na^+^/Ca^2+^ exchanger cooperate with the mitochondria and the sarco/endoplasmic reticulum Ca^2+^ ATPases (SERCA) (Berridge, 2008).

SERCA proteins are expressed mainly as three isoforms SERCA1, 2, and 3. SERCA2 is the most widely distributed isoform and plays a central role in Ca^2+^ homeostasis in cardiovascular tissues. The *ATP2A2* gene gives rises to different SERCA2 variants through alternative splicing. SERCA2a, often called the cardiac isoform, is expressed in cardiac muscle, slow-twitch skeletal muscle and SMC, while SERCA2b displays ubiquitous expression in muscle and non-muscle cells. SERCA2a and SERCA2b are produced by alternative splicing and only differ by replacement of the last 4 amino acids of SERCA2a by 49 additional amino acids in SERCA2b. However, both SERCA2 isoforms display different functionality with SERCA2b presenting a two times higher affinity for Ca^2+^, but a twofold lower catalytic turnover rate compared to SERCA2a (Lipskaia et al., 2013, 2014; McIvor et al., 2018).

In the heart, SERCA2a is the prominent isoform and SERCA2a is down-regulated during pathological end stage heart failure. Down-regulation of SERCA2a is associated with impaired intracellular Ca^2+^ handling in these pathologies, whereas restoration of SERCA2a expression by gene-transfer improved contractility in preclinical models of heart failure (Kho et al., 2012). Clinical trials in heart failure patients with SERCA2a adenoviral gene therapy initially showed promising results (CUPID) (Jessup et al., 2011), but follow-up trials (CUPID-2 and AGENT-HF) failed to demonstrate benefit of SERCA2a gene transfer, most likely due to inadequate gene transfer (Greenberg et al., 2016; Hulot et al., 2017).

In the vasculature, both SERCA2a and 2b are expressed. In vascular SMC from the rat aorta, SERCA2a and SERCA2b mRNA accounted for respectively 30 and 70% of total SERCA2 mRNA (Levitsky et al., 1993). Paradoxically, another study reported more abundance of the SERCA2a isoform in mouse aorta (Lipskaia et al., 2014). The specific roles of SERCA2a and 2b in vascular tissue, however, are not fully understood. There are indications that SERCA2b, among others, controls cell cycle and proliferation, whereas SERCA2a expression is more related to differentiation-specific contractile activity (Lipskaia et al., 2005, 2013).

Some information on the physiological role of the different SERCA isoforms was obtained from gene ablation studies in mice. A homozygous knock-out of SERCA2 is embryonic lethal, whereas in heterozygous knock-out mice with only one functional SERCA2 allele, SMC contractile behavior was unchanged (Ishida and Paul, 2005). Additionally, a mouse model was developed in which the SERCA2-encoding gene (*Atp2a2*) was modulated such that the alternative splicing underlying the expression of SERCA2a is prevented. In these mice, the SERCA2a isoform is replaced by SERCA2b (referred to as SERCA2^b/b^ mice, as compared to wild-type SERCA2^a/b^ mice where both SERCA2 variants are normally expressed), which resulted in a clear cardiac structural and functional phenotype (Ver Heyen et al., 2001; Vangheluwe et al., 2006). Adult SERCA2^b/b^ animals developed a mild compensatory concentric cardiac hypertrophy with impaired cardiac contractility and relaxation, but preserved β-adrenergic response. Cardiac homogenates displayed reduced Ca^2+^ uptake levels and in isolated cells, relaxation and Ca^2+^ removal by the SR were significantly reduced. Because the SERCA2a to SERCA2b swap not only occurs in cardiac tissue, but probably also in other tissues, the present study investigated changes in vascular reactivity of aorta segments of SERCA2^a/b^ and SERCA2^b/b^ mice.

## Materials and Methods

### Mice

The studies were approved by the Ethical Committee of the University of Antwerp, and the investigations conform with the Guide for the Care and Use of Laboratory Animals published by the US National Institutes of Health (NIH Publication No. 85-23, revised 1996). Generation of the mouse strain is described in Ver Heyen et al. (2001). SERCA2^b/b^ mice and their corresponding wild-type strain SERCA2^a/b^ were used at the age of 4 to 5 months (SERCA2^a/b^: *n* = 17, 13 females, 4 males, age 140 ± 6 days, body weight: 29.7 ± 1.1 g; SERCA2^b/b^, *n* = 18, 13 females, 5 males, age 142 ± 5 days, body weight 28.1 ± 0.9 g).

### Aortic Segments

After anesthesia (sodium pentobarbital, 75 mg kg^–1^, i.p.), the thoracic aorta was carefully removed, stripped of adherent tissue and dissected systematically. Starting 3 mm from the origin of the left subclavian artery (where the azygos vein crosses the aorta) down to the diaphragm [11], the descending thoracic aorta was cut in segments of 1 mm (Ca^2+^-force studies) or 2 mm (vasomotor studies) width.

### Studies of SMC Function

In segments for studying SMC function, NO formation was inhibited with 300 μM L-NAME and 10 μM indomethacin was present throughout the experiments to avoid any vasomotor interference due to prostanoids (Okon et al., 2002). Segments of 2 mm width were mounted between two parallel tungsten wire hooks in 10 ml organ baths. Isometric tension was measured with a Statham UC2 force transducer (Gould) connected to a data acquisition system (Powerlab 8/30, ADInstruments). Vessels were immersed in Krebs-Ringer solution (37°C, 95% O_2_/5% CO_2_, pH 7.4) with (in mM): NaCl 118, KCl 4.7, CaCl_2_ 2.5, KH_2_PO_4_ 1.2, MgSO_4_ 1.2, NaHCO_3_ 25, CaEDTA 0.025 and glucose 11.1 and gradually stretched until a stable loading tension of 16–20 mN was attained.

When SMC cytosolic Ca^2+^ and force measurements were to be combined, ECs were removed by perfusion of the aorta with 3 ml 0.01% Triton X 100 in Krebs–Ringer solution, as described (Van Assche et al., 2007), or mechanically by rubbing the interior of the segment with a braided silk wax. Endothelium-denuded segments were mounted in a wire (40 μm) myograph (Danish Myotechnology A/S, Aarhus, Denmark) above an inverted microscope, immersed in Krebs-Ringer solution (37°C) and continuously aerated with 95% O_2_/5% CO_2_ (pH 7.4). Only segments, in which endothelium-dependent relaxation by acetylcholine or ATP was absent, were considered as EC denuded. The segment was loaded *in situ* with 10 μM Fura-2 AM in Krebs-Ringer solution with 0.02% Pluronic for 120 min at room temperature. Then, the segment was set to its normalized diameter at 13.3 kPa according to Mulvany and Halpern (1977). The single emission (510 nm) ratio at dual excitation (340 and 380 nm) was used as a relative measure of free [Ca^2+^]_*i*_ (relative units, RU) and was analyzed with Felix software (PTI, United States). At the end of the experiment 2 mM MnCl_2_ was added to the segment to determine background emission values, which were subtracted from the respective emission values during the experiment. Absolute levels of [Ca^2+^]_*i*_ are not reported due to the uncertainty of the conventional calibration method in intact tissues (Tosun et al., 1998; Lalli et al., 1999). Contractile force was measured simultaneously and reported in mN mm^–1^.

### Studies of EC Function

When only vasomotor responses were studied, aorta rings (2 mm) were mounted as described above, but the L-NAME was omitted and only indomethacin 10 μM was added to the Krebs-Ringer solution to avoid any interferences by prostanoids. When isometric force and EC Ca^2+^ measurements were to be combined, the segments were mounted and loaded as described above, but without chemical or mechanical EC removal.

### Real-Time RT-PCR

Mice were anesthetized with sodium pentobarbital (Nembutal^§^, 75 mg kg^–1^, i.p.). The aorta was carefully excised and cleaned from adherent tissue. The aorta was rinsed with 10 ml Krebs-Ringer solution ([mM] NaCl 118, KCl 4.7, CaCl_2_ 2.5, KH_2_PO_4_ 1.2, MgSO_4_ 1.2, NaHCO_3_ 25, CaEDTA 0.025 and glucose 11.1) and then cut in small pieces, which were placed in 350 μl Trizol reagent (Invitrogen, Merelbeke, Belgium). Subsequently, RNA was purified using the RNeasy MinElute kit (Qiagen, Antwerp, Belgium) and collected in 25 μl. RNA quality was tested by electrophoresis using the Agilent Bioanalyzer. All RNA samples had a RNA integrity number (RIN) between 7.5 and 7.7. Relative expression of genes of interest was evaluated using the Two Step RT qPCR Core kit (Eurogentec, Seraing, Belgium). RNA was converted into cDNA (Reverse Transcriptase Core kit, Eurogentec, Seraing, Belgium), then subjected to quantitative PCR (qPCR Core kit, Eurogentec) on an ABI 7300 Instrument (Applied Biosystems, Foster City, CA, United States) using the following primers and probes: SERCA2A: Forward Primer: CACTTCTTGATCCTCTACGTGGAA; Reverse Primer: TACTCCAGTATTGCGGGTTGTTC; Probe: 6-FAM-CAACCCGCAATACT-MGB; SERCA2B: Forward Primer: CACTTCTTGATCCTCTACGTGGAA; Reverse Primer: CAGGCTGCACACACTCTTTACC; Probe: 6-FAM-CAACCCGGTAAAGAG-MGB. Relative expression of mRNA species was calculated using the comparative threshold cycle (Ct) method using β-actin (ACTB) as reference genes; the mean value of the six CTA extracts was set to 1 using the 2^–ΔΔCt^ method.

### Western Blot

For each mouse genotype, five isolated aortic ring samples were combined. The frozen tissues were grinded with pestle and mortar in liquid nitrogen, then resuspended in 500 μl RIPA buffer (Pierce) supplemented with SIGMAFAST protease inhibitor (Merck). Further homogenization was done by douncing each sample 50 strokes on ice. The resultant suspension was rotated in head over head for 3 h at 4°C, then centrifuged at 14000 rpm to remove the pellet. The protein concentration of the supernatant was determined by Bradford method.

For immunoblotting, 10 μg cell lysate of 2a/b and 2b/b samples were separated via NuPAGE 4–12% Bis-Tris polyacrylamide gel electrophoresis at 200 V, 45 min in MOPS buffer (Thermo Fisher Scientific), then transferred to PVDF membrane (100 V, 1 h) in transfer buffer (Thermo Fisher Scientific). Primary antibodies against SERCA2a (1:10000, R15-7, homemade), SERCA2b (1:10000, R5-24, homemade), total SERCA (1:2000, SB49, homemade), β-actin (1:2000, Monoclonal Anti-β-Actin, Merck), and horse radish peroxidase linked secondary antibodies (Cell Signaling Technology) were used for enhanced chemiluminescent detection (Bio-rad ChemiDoc). The intensities of the bands were quantified with Image Lab.

### Immunostaining

Aortic and left ventricular heart segments of SERCA2a/b and SERCA2b/b mice were collected immediately after dissection, embedded in NEG-50TM (Thermo Scientific) and kept at −80°C. Cryosections of the aortic segments (± 6 μm) were air-dried for 20 min and, then, fixated with acetone (5 min). The cryostat sections were rinsed with phosphate buffered saline (PBS, 5 min) at room temperature. Following incubation overnight at 4°C with the primary antibodies against SERCA2a (1:10000, R15-7, homemade), SERCA2b (1:10000, R5-24, homemade) and 3 times wash (10 min) with PBS, they were incubated for 2 h at 37°C with secondary antibody (goat anti-rabbit Alexa green, Molecular Probes). The cryostat sections were placed again in washing buffer (15 min), washed 3 times (5 min) in PBS and mounted in VECTASHIELD Antifade Mounting Medium with DAPI (nuclear staining). Images were made on an Olympus BX40 epifluorescence microscope with a SensiCam 12 bit cooled CCD (PCO, Germany) and stored on computer for later analysis with Photoshop software.

### Data Analysis

All results are expressed as mean ± SEM; n represents the number of mice, s the number of segments. Dose-response curves were fitted with sigmoidal dose-response equations with variable slope, which revealed maximum contraction or relaxation (E_max_) and the logarithm of the concentration resulting in 50% of the maximal response (log EC_50_) for each vessel segment. Two-way ANOVA with Bonferroni post-test and unpaired *t*-test were performed using GraphPad Prism, version 6 (GraphPad Software, San Diego, CA, United States) and were used to compare means of the different experimental groups. A 5% level of significance was selected.

### Materials

Sodium pentobarbital (Nembutal) was obtained from Sanofi (Brussels, Belgium), indomethacin from Federa (Belgium), antibody against CD31 from BD (Erembodegem, Belgium), phenylephrine hydrochloride, acetylcholine-chloride, LNNA, L-NAME, Na_2_-ATP from Sigma, Fura 2-AM from Molecular Probes (The Netherlands), trinitroglycerin solution (1%, Merck, Belgium), CPA (Tocris Bioscience, United Kingdom).

## Results

### SERCA2a/SERCA2b mRNA Transcripts and Protein Expression

**FIGURE 1:**
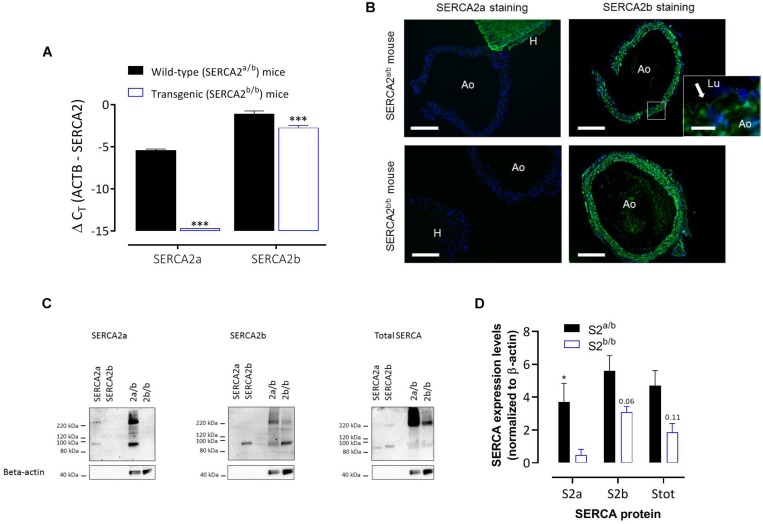
SERCA2 expression in the aorta of SERCA2^a/b^ and SERCA2^b/b^ mice. **(A)** Real-time RT-PCR on RNA isolated from aortic segments of SERCA2^a/b^ mice and SERCA2^b/b^ mice. SERCA2a and SERCA2b transcripts were determined versus the reference gene transcript β-actin (ACTB) (*n* = 3). **(B)** Immunofluorescent staining of sections of thoracic aorta (Ao) of SERCA2^a/b^ (upper row) and SERCA2^b/b^ (lower row) mice (*n* = 3) with antibodies against SERCA2a (left column) and SERCA2b (right column). For SERCA2a, sections of left heart ventricle (H) are included. (Lu) indicates lumen of the aorta. SERCA2 was stained green, whereas the nuclei were stained blue with DAPI. A 10 times larger image of the aortic wall for SERCA2a is also shown for the SERCA2^a/b^ mouse aorta (insert) with the arrow indicating SERCA2b staining of ECs. Horizontal scale bar is 320 μm (30 μm for the insert). **(C)** Representative examples of Western blot for the SERCA2a isoform (left, *n* = 4), SERCA2b isoform (middle, *n* = 5) and total SERCA (right, *n* = 3) in aortic segments of SERCA2^a/b^ (S2a/b) and SERCA2^b/b^ (S2b/b) mice (last two lanes) with the respective peptides as positive controls (SERCA2a and SERCA2b, first two lanes). **(D)** Summary of the WB data (*n* = 3–5), normalized to β-actin. *, ***p < 0.05, 0.001 SERCA2^b/b^ versus SERCA2^a/b^ mice.

Real-time RT-PCR on RNA isolated from aortic segments of SERCA2^a/b^ and SERCA2^b/b^ mice (Figure 1A) revealed the presence of SERCA2a and SERCA2b mRNA transcripts in the aorta of SERCA2^a/b^ mice with about 20-fold higher SERCA2b levels compared to SERCA2a (assuming an amplification efficiency close to 100%). SERCA2a mRNA was undetectable in SERCA2^b/b^ mice, confirming successful prevention of alternative splicing of the modified SERCA2 allele to SERCA2a. SERCA2b transcripts were significantly lower in SERCA2^b/b^ than in SERCA2^a/b^ mouse aorta.

Immunohistochemistry (IHC) using antibodies against SERCA2a and SERCA2b protein isoforms confirmed the mRNA data and revealed that in SERCA2^a/b^ mouse aorta the SERCA2a protein levels were much lower in aorta than in cardiac tissue (Figure 1B). SERCA2a staining was clearly present in hearts of SERCA2^a/b^ mice, but was absent from hearts of SERCA2^b/b^ animals. SERCA2b staining was present in ECs and SMCs, and was similarly distributed in SERCA2^a/b^ and SERCA2^b/b^ animals. Western blot analysis (Figures 1C,D) confirmed the data of the RT-PCR and IHC in that the SERCA2a protein isoform was present in SERCA2^a/b^ mice, but absent from SERCA2^b/b^ animals. In line with previous observations in the heart of these mice (Ver Heyen et al., 2001), SERCA2b displayed about 1.6 times higher expression in SERCA2^a/b^ than in SERCA2^b/b^ aortic segments.

### Aortic Dimensions of SERCA2^a/b^ and SERCA2^b/b^ Mice

Aortic diameter and wall thickness were not different between SERCA2^a/b^ and SERCA2^b/b^ mice (respectively 829 ± 31 μm versus 789 ± 47 μm for diameter and 140 ± 13 μm versus 134 ± 13 μm for wall thickness, *n* = 5, *p* > 0.05, Supplementary Figure S1).

### Contractile Responses

Contractions were elicited by means of two stimuli, which involve different mobilization of intra- and extracellular Ca^2+^. Depolarization with high external K^+^ causes isometric contraction of aortic segments by opening voltage-gated Ca^2+^ channels only (Fransen et al., 2012). α_1_-Adrenoceptor stimulation with phenylephrine (PE), on the other hand, causes phasic contractions by release of intracellular Ca^2+^ from the SR Ca^2+^ stores and tonic contractions through Ca^2+^ influx via voltage-gated Ca^2+^ channels and non-selective cation channels (Fransen et al., 2015). By comparing these contractile stimuli in SERCA2^a/b^ and SERCA2^b/b^ mice, the functional role of SERCA2a/b in aortic vascular smooth muscle could be explored.

#### Depolarization

**FIGURE 2:**
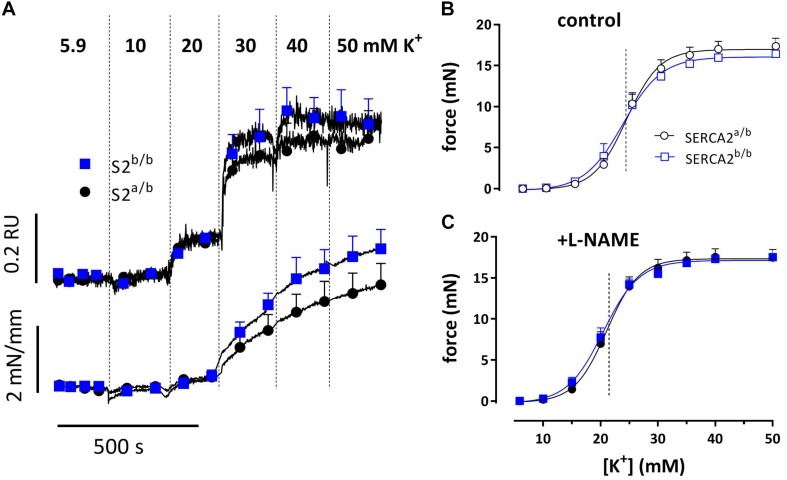
Intracellular Ca^2+^ signals and concomitant force development induced by stepwise increase of K^+^ in aortic segments of SERCA2^a/b^ and SERCA2^b/b^ mice. Sensitivity of SERCA2^a/b^ (circles) and SERCA2^b/b^ (squares) aortic segments to depolarization with elevated external K^+^. **(A)** Ca^2+^ signals and concomitant force development with increasing concentrations of extracellular K^+^, measured in Fura-2-loaded endothelium-denuded aortic segments mounted in a myograph (*n* = 4). Force at increasing K^+^ for endothelium-intact aortic segments measured in an isometric organ bath in control **(B)** and after inhibition of eNOS with 300 μM L-NAME **(C)** (**B,C**: *n* = 5).

Basal SMC Ca^2+^ levels of 1.04 ± 0.04 RU for SERCA2^*a*/b^ and 1.02 ± 0.05 RU for SERCA2^*a*/b^ (*n* = 14) were not different. Depolarization of the aortic segments with stepwise elevation of K^+^ concentrations from 5.9 to 10, 20, 30, 40, and 50 mM K^+^ elicited parallel Ca^2+^ signals and contractions, which were not significantly different between the strains with or without supplementation of L-NAME, an inhibitor of basal NO release (Figure 2).

#### α_1_-Adrenergic Stimulation of Aortic Segments With 1 μM Phenylephrine

**FIGURE 3:**
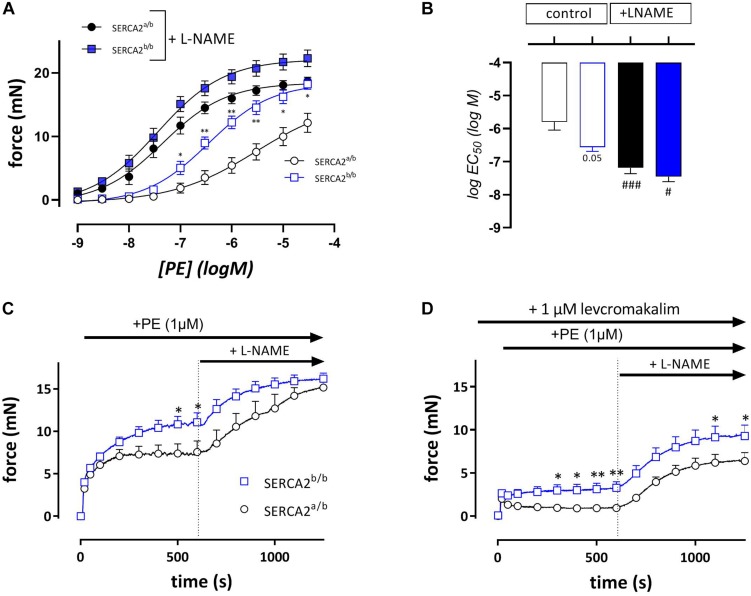
Isometric contractions by PE are higher in aortic segments of SERCA2^b/b^ compared to SERCA2^a/b^**.** Concentration-effect curves **(A)** and respective logEC_50_ values **(B)** for contractions by PE (10^– 9^ to 3*10^– 5^ M) in the absence (open symbols) and presence (closed symbols) of 300 μM L-NAME in SERCA2a (circles) and SERCA2b (squares) aortic segments. Graphs show the contraction induced by 1 μM PE in the absence **(C)** and presence **(D)** of 1 μM levcromakalim. 10 Min after the addition of PE, 300 μM L-NAME was added in all conditions to inhibit release of basal NO. *, **p < 0.05, 0.01 SERCA2^b/b^ versus SERCA2^a/b^ (*n* = 5); #, ###*p* < 0.05, 0.001 + L-NAME versus –L-NAME.

In endothelium-intact segments, phenylephrine (PE, 1 nM- 30 μM) caused significantly higher contractions in SERCA2^b/b^ than in SERCA2^a/b^ mice with a near-significant shift of the concentration response curve to the left (Figures 3A,B). After addition of 300 μM L-NAME to inhibit basal NO release, isometric force increased more in the SERCA2^a/b^ than in the SERCA2^b/b^ segments with significant shifts in PE-sensitivity for both strains (Figure 3B). At steady-state the isometric contractions were not significantly different between the strains. When these experiments were repeated with a single concentration of PE (1 μM) in the absence (Figure 3C) or presence (Figure 3D) of 1 μM levcromakalim, contractions were larger in SERCA2^b/b^ than of SERCA2^a/b^ segments both in the absence and presence of L-NAME (Figure 3C). In the presence of levcromakalim, which prevents the activation of L-type Ca^2+^ influx by hyperpolarization of the aortic segments, contraction of vascular SMC is mediated by non-selective cation influx only (Fransen et al., 2015), suggesting increased Ca^2+^ influx in SERCA2^b/b^ aortic segments stimulated with PE.

#### Phasic Contractions by PE in the Presence of 0 μM Ca^2+^

**FIGURE 4:**
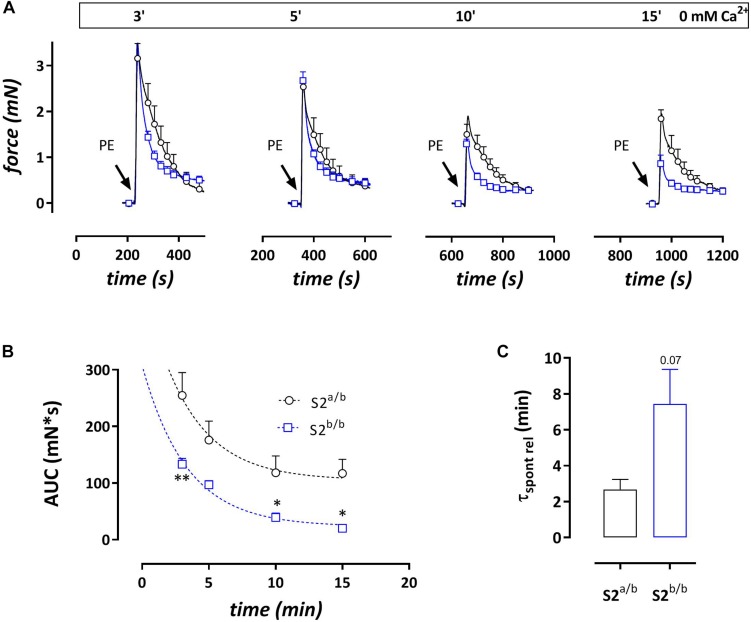
Phasic contractions by PE (1 μM during 0 mM Ca^2+^ challenge) display slower and smaller spontaneous SR contractile Ca^2+^ release but always faster relaxation kinetics in SERCA2^b/b^ segments. **(A)** Phasic contractions by PE (1 μM) upon 0 mM Ca^2+^ challenge of various duration decrease with duration of the 0 mM Ca^2+^ challenge, but at each time interval display consistently faster relaxation kinetics in SERCA2^b/b^ than in SERCA2^a/b^ segments. **(B)** The AUC of the phasic contraction, representative for the SR Ca^2+^ store content, is significantly lower, but more complete in SERCA2^b/b^ segments. **(C)** The decline of the AUC of the phasic contraction over different durations of 0 mM Ca^2+^ challenges tended to be slower in SERCA2^b/b^ segments. *p < 0.05, SERCA2^b/b^ (S2^b/b^) versus SERCA2^a/b^ (S2^a/b^), (*n* = 4–5).

Addition of 1 μM PE in the absence of external Ca^2+^ caused phasic contractions which decreased with duration of the challenge with 0 mM Ca^2+^, suggesting spontaneous emptying of the contractile SR Ca^2+^ stores in the absence of extracellular Ca^2+^ (Leloup et al., 2015) (Figure 4). With each duration of the 0 mM Ca^2+^ period (3, 5, 10, or 15 min) the AUC of the phasic contractions was consistently lower in SERCA2^b/b^ segments (Figure 4B) and in these segments spontaneous emptying of the contractile SR Ca^2+^ stores occurred slower (7.4 ± 1.9 versus 2.7 ± 0.6 min, *p* = 0.07, *n* = 5–4, Figure 4C) and was more complete. Subsequent phasic contractions by PE in other experiments were measured after a period of 3 min challenge with 0 mM Ca^2+^. Interestingly, and as shown in Figure 4A, at each interval after addition of 0 mM Ca^2+^, PE-induced phasic contractions showed a faster decay in SERCA2^b/b^ compared to SERCA2^a/b^ segments and at 3 min the time constants were 17 ± 1 s versus 39 ± 4 s, *p* < 0.001 (*n* = 5). The time constants of force development were not different (respectively 2.3 ± 0.1 and 2.1 ± 0.1 s).

#### Tonic Contractions by PE Upon Re-addition of 3.5 mM Ca^2+^

**FIGURE 5:**
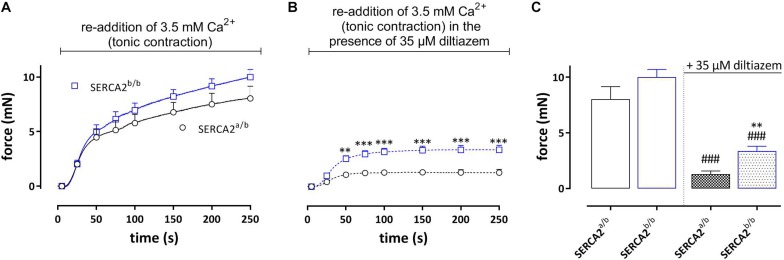
Tonic contractions by PE (upon re-addition of 3.5 mM Ca^2+^) in SERCA2^a/b^ and SERCA2^b/b^ segments. **(A)** Following the phasic contractions, tonic contractions by PE (1 μM) were determined in SERCA2^a/b^ and SERCA2^b/b^ mouse aortic segments by re-addition of 3.5 mM Ca^2+^. Tonic contractions by PE tended to be higher in SERCA2^b/b^ than in SERCA2^a/b^ segments. **(B)** In the presence of 35 μM diltiazem tonic contractions by PE were higher in SERCA2^b/b^ than in SERCA2^a/b^ segments. **(C)** Amplitudes of contractions after 250 s in the absence and presence of diltiazem. **, ###*p* < 0.01, 0.001 SERCA2^b/b^ versus SERCA2^a/b^, ###: diltiazem versus control (*n* = 5).

Re-addition of external Ca^2+^ after the phasic contraction induced a tonic contraction (Figure 5), which was slightly higher in SERCA2^b/b^ aortic segments. Following inhibition of L-type Ca^2+^ channels with 35 μM diltiazem, the remaining contraction, which is now due to Ca^2+^ influx via non-selective cation channels (NSCC) was significantly higher in SERCA2^b/b^ segments (Figures 5B,C).

#### Refilling of SR Ca^2+^ Stores After α_1_-Adrenergic Stimulation With 1 μM PE

**FIGURE 6:**
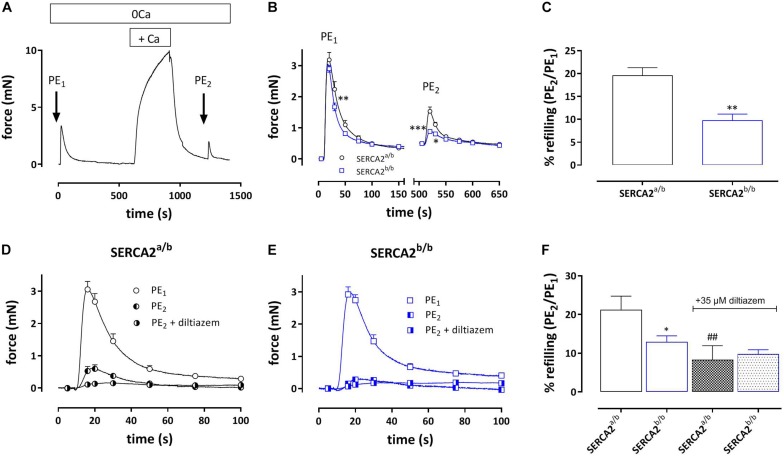
Amount of re-filling of the PE-sensitive contractile SR Ca^2+^ stores in SERCA2^a/b^ and SERCA2^b/b^ segments. **(A)** Experimental protocol to investigate PE (1 μM)-sensitive phasic contractions in 0 mM Ca^2+^ after a period of 5 min of Ca^2+^ re-addition (+ Ca^2+^) to refill the SR Ca^2+^ stores. **(B)** Phasic contractions of the first (PE_1_) and second (PE_2_) phasic contraction by 1 μM PE in 0 mM Ca^2+^ in SERCA2^a/b^ (black) and SERCA2^b/b^ (blue) aortic segments. **(C)** Ratio of AUC of the first and second PE application (PE1/PE2 in%) in both strains. When after PE1 35 μM diltiazem was added, refilling was attenuated in SERCA2^a/b^ segments **(D)**, but was less affected in SERCA2^b/b^ segments **(E)**. **(F)** Ratio of AUC of the first and second PE application (PE1/PE2 in%) in control (open bars) and after addition of 35 μM diltiazem (patterned bars) in both strains. *, **, ***p < 0.05, 0.01, 0.001 SERCA2^b/b^ versus SERCA2^a/b^ (*n* = 5), ##*p* < 0.01 diltiazem versus control (*n* = 5).

During the Ca^2+^ re-addition phase, SR Ca^2+^ stores, which were emptied by admission of 1 μM PE, are re-filled, mainly by activity of L-type Ca^2+^ channels (Leloup et al., 2015). By measuring the second phasic contraction during the re-filling of the stores, the amount of re-filling can be estimated. Figure 6A shows the experimental protocol, whereas Figure 6B shows the first and second PE-induced phasic contraction. In SERCA2^a/b^ aortic segments the second PE application caused a phasic contraction with an AUC which was about 20% of the first PE response (Figure 6C). In SERCA2^b/b^ segments the contractile SR store was re-filled for only about 10%. When 35 μM diltiazem was added after PE_1_, phasic contractions were significantly reduced in SERCA2^a/b^ aortic segments (Figure 6D), but not in SERCA2^b/b^ segments (Figures 6E,F).

#### Contractions by SERCA Inhibition With CPA

**FIGURE 7:**
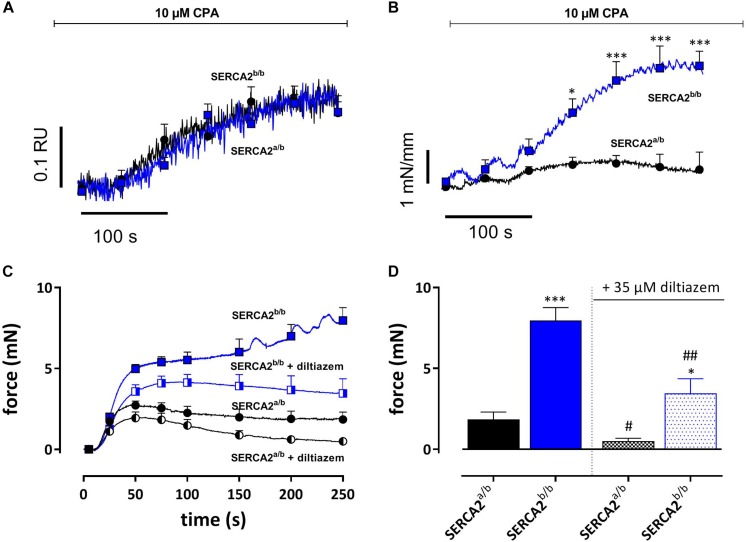
SERCA Ca^2+^ re-uptake inhibition with CPA caused similar intracellular Ca^2+^ signals, but increased isometric force signals in SERCA2^b/b^ more than SERCA2^a/b^ aortic segments. **(A)** Intracellular Ca^2+^ and **(B)** isometric force were simultaneously measured in endothelium-denuded (1 mm width) aortic segments (*n* = 5) of SERCA2^a/b^ and SERCA2^b/b^ mice when treated with 10 μM CPA as indicated by the horizontal bar. *, ***p < 0.05, 0.001 SERCA2^b/b^ versus SERCA2^a/b^. **(C,D)** Tonic contractions by 10 μM CPA were measured in (2 mm width) aorta segments treated with 300 μM L-NAME to inhibit eNOS production. CPA-induced contractions were increased in SERCA2^b/b^ also after inhibition of L-type Ca^2+^ influx with diltiazem. CPA was added during a 0 mM Ca^2+^ challenge, while re-addition of 3.5 mM Ca^2+^ (+ CPA solution) resulted in a contractile response, both in the absence and presence of 35 μM diltiazem. *, **, ***p < 0.05, 0.01, 0.001 SERCA2^b/b^ versus SERCA2^a/b^, #, ##*p* < 0.05, 0.01 diltiazem versus control (*n* = 5).

The SERCA inhibitor CPA (10 μM) causes large intracellular Ca^2+^ transients in SMC without producing substantial contractions (Fransen et al., 2015). In the following experiments SERCA was inhibited in EC-denuded aortic segments of SERCA2^a/b^ and SERCA2^b/b^ mice to investigate intracellular Ca^2+^ and concomitant isometric contractions. Addition of 10 μM CPA caused a large increase of internal Ca^2+^ (0.27 ± 0.05 RU) in SERCA2^a/b^ aortic segments, which was paralleled by a small and transient increase of isometric force (E_max_ 0.70 ± 0.23 mN/mm) (Figures 7A,B). In SERCA2^b/b^ segments, the Ca^2+^ signal was not different (0.27 ± 0.03 RU), but was accompanied by a substantially higher isometric force development (3.60 ± 0.43 mN/mm) (Figures 7A,B). These data were confirmed in aortic segments, in which endothelial NO release was inhibited with 300 μM L-NAME (Figure 7C). SERCA inhibition with 10 μM CPA caused significantly higher contractions in SERCA2^b/b^ than in SERCA2^a/b^ segments (Figures 7C,D). After inhibition of L-type Ca^2+^ channels with 35 μM diltiazem, the contraction by CPA was inhibited but the difference between the two strains remained.

### Relaxation of PE-pre-constricted Segments by NO and cGMP

To assess endothelium-dependent and endothelium-independent effects of NO or cGMP in vascular SMCs, PE-constricted segments were exposed to sources of endogenous NO (acetylcholine, ATP, CPA), exogenous NO (${\text{NO}}_{2}^{-}$, DEANO) and the membrane permeable cyclic GMP-analog 8-Br-cGMP (Table 1). Except for ATP, there was no difference between endothelium-dependent or endothelium-independent relaxation of pre-constricted aortic segments of SERCA2^a/b^ and SERCA2^b/b^ mice.

**TABLE 1:** Endogenous NO, exogenous NO and cGMP cause relaxation of aortic segments of SERCA 2^a/b^ and SERCA2^b/b^ segments, which were pre-contracted with 1 μM PE.

	SERCA2^a/b^		SERCA2^b/b^		Number of mice
	log(EC_50_) (logM)	E_max__(%)_	log(EC_50_) (logM)	E_max__(%)_	
ACh	−6.67 ± 0.18	58 ± 10	−6.62 ± 0.13	59 ± 7	9, 9
ATP	−5.09 ± 0.12	109 ± 5	−5.90 ± 0.09*	113 ± 2	8, 8
CPA	−6.96 ± 0.11	96 ± 4	−7.00 ± 0.16	100 ± 7	8, 8
${\text{NO}}_{2}^{-}$	−6.25 ± 0.08	119 ± 4	−6.44 ± 0.10	115 ± 3	4, 4
DEANO	−7.02 ± 0.24	100 ± 2	−7.22 ± 0.13	99 ± 2	10, 10
8-Br-cGMP	−5.79 ± 0.04	87 ± 2	−5.83 ± 0.07	85 ± 3	4, 4

*Concentration-relaxation curves for endogenous NO release with ACh, ATP and CPA, exogenous NO supply with $N\,O_{2}^{-}$ or DEANO and the membrane permeable cGMP analog, 8-Br-cGMP, revealed log(EC_50_) and maximal relaxation (E_*max*_) values as indicated in the table. For relaxation by$N\,O_{2}^{-}$, DEANO and 8-Br-cGMP segments were treated with 300 μM L-NAME to inhibit basal endothelial release of NO. ^∗^*p* < 0.05 SERCA2^*b/b*^ versus SERCA2^*a/b*^.*

**FIGURE 8:**
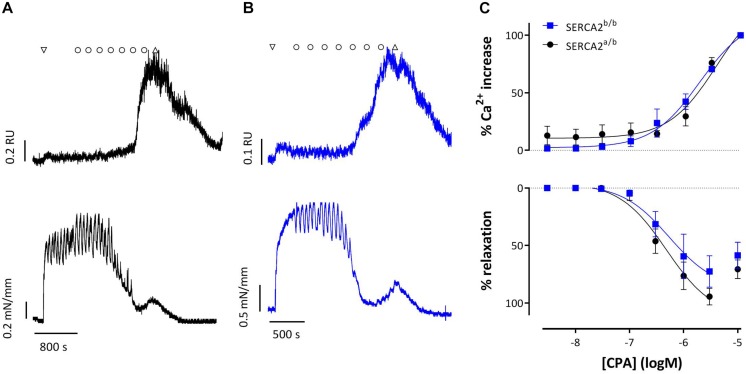
Intracellular Ca^2+^ signals and concomitant force development induced by CPA in endothelium-intact aortic segments of SERCA2^a/b^ and SERCA2^b/b^ mice stimulated with 1 μM PE. **(A,B)** Representative example of intracellular Ca^2+^ (upper row) and concomitant force (lower row) upon addition of 1 μM PE (down triangle), CPA (10^–8^–10^–5^ M, circles) and wash (up triangle) in a SERCA2^a/b^ segment (black, **A**) and SERCA2^b/b^ segment (blue, **B**). **(C)** % increase of Ca^2+^ (top) and % relaxation as a function of [CPA].

Figure 8 shows representative examples of simultaneous endothelial Ca^2+^ signals and isometric contraction/relaxation of aortic segments with intact endothelium. 1 μM PE caused a very small increase of intracellular Ca^2+^, which is probably due to the Ca^2+^ increase in SMC and which suggests rather selective loading of EC with Fura 2-AM. CPA (0.3 μM and higher) caused a concentration-dependent increase of endothelial intracellular Ca^2+^, which was accompanied by relaxation of PE-pre-contracted segments in both strains. Only the highest concentration of 10 μM CPA caused contraction due to increase of Ca^2+^ in the SMC. Also in this experimental setting, CPA-induced Ca^2+^ (EC_50_ −5.44 ± 0.17 logM to −5.75 ± 0.10 logM) and relaxation (EC_50_ −6.32 ± 0.15 logM to −6.27 ± 0.26 logM) were unaltered in SERCA2^b/b^ segments.

## Discussion

According to several reports, vascular EC express two of the three SERCA genes, i.e., SERCA2 (SERCA2b) and SERCA3 (SERCA3a) (Mountian et al., 1999; Khan et al., 2000), whereas vascular SMC mainly express the spliced isoforms SERCA2a and 2b (Le Jemtel et al., 1993; Clark et al., 2010; Lipskaia et al., 2014). In cardiac muscle, SERCA2a is the prominent SERCA isoform (95%). In SMC of pig aorta, 72% of the SERCA2 mRNAs encode the SERCA2b isoform (Eggermont et al., 1990). Also in rat aorta, SERCA2b is the more abundant SERCA isoform (Levitsky et al., 1993). Similarly, the real-time PCR data of the current study points toward a significantly higher mRNA expression of the SERCA2b isoform in the aorta of SERCA2^a/b^ mice. The mRNA data were confirmed by IHC and Western blot. SERCA2b was abundantly present in the aorta, while SERCA2a was hardly detected by IHC or Western blot as compared to cardiac tissue. Conversely, Lipskaia et al. (2014) reported a high mRNA expression of SERCA2a (91% of total SERCA) and low expression of SERCA2b (∼5%) in the aorta of C57Bl/6 mice. Possible explanations for this discrepancy are genetic background (C57Bl/6 versus 50/50 R1 129/Swiss), age of the mice (8 weeks versus 4/5 months), aortic segment origin and RNA quantification methods.

Here, we investigated vascular reactivity in the SERCA2^b/b^ mouse model. With this model, it has been shown that SERCA2a is essential for normal cardiac performance (Ver Heyen et al., 2001). SERCA2a-isoform deficiency leads to increased incidence of fetal/neonatal mortality and cardiac structural malformations. Hearts of SERCA2^b/b^ mice develop hypertrophy and display decreased total SERCA2 mRNA and protein levels (50%). Concomitantly, impaired cardiac contractility and relaxation occurred with reduced SR Ca^2+^-uptake activity (−50%). The apparent Ca^2+^ affinity of the Ca^2+^ uptake was increased in SERCA2^b/b^, suggesting a higher apparent Ca^2+^ affinity for SERCA2b in cardiac homogenates of SERCA2^b/b^ mice. A prominent role of SERCA2a in vascular tissue was not evident. In a heterozygous SERCA2 knock-out mouse (SERCA2^±^, 50% reduction of SERCA2), aortic contractility to PE or depolarization (KCl) was not changed, and there was no difference in relaxation to activation of the A-kinase pathway (forskolin) or G-kinase pathway (sodium nitroprusside) (Ishida and Paul, 2005). In the present study, however, despite the small amount of SERCA2a versus SERCA2b in the mouse aorta, functional effects of SERCA2a ablation were considerable. Importantly, also a decrease in SERCA2b mRNA and protein (about 60%) expression in the aorta of SERCA2^b/b^ mice was observed, which may, in part, contribute to the observed vascular phenotype.

Depolarization of aortic segments with increased extracellular K^+^ induced similar Ca^2+^ signals and isometric force in SERCA2^b/b^ and in SERCA2^a/b^ animals. We have previously shown that isometric contractions of C57Bl6 aortic segments upon depolarization are completely due to fast and slow Ca^2+^ influx via voltage-gated Ca^2+^ channels (VGCC) (Fransen et al., 2012). Hence, the results of the present study suggest that the contractile SR (SERCA2a and SERCA2b) does not contribute to depolarization-induced contractions in mouse aorta. Hence, in SERCA2^b/b^ mice, there seems to be no functional change of the L-type Ca^2+^ channel mediated contractions as long as the SR is not involved in the contraction.

However, prominent differences in contractile behavior were observed with contractions elicited by α_1_ adrenergic stimulation with PE. These contractions occur in two phases. First, PE induces a transient, phasic contraction, which is due to Ca^2+^ release from IP_3_-sensitive SR Ca^2+^ stores. This phasic contraction depends on the SR Ca^2+^ store content and the ratio of Ca^2+^ release from the stores and removal of Ca^2+^ from the cytoplasm via plasmalemmal Ca^2+^ pumps (PMCA) (Leloup et al., 2015). The initial phasic contraction is followed by a persistent, tonic contraction, which is due to Ca^2+^ influx via VGCC and via NSCC (Fransen et al., 2015). Interestingly, when the VGCC Ca^2+^ influx of the PE contraction was prevented by hyperpolarizing vascular SMCs with levcromakalim, which is an ATP-dependent K^+^ channel agonist, the difference in contraction between SERCA2^a/b^ and SERCA2^b/b^ mice remained. This observation suggests an altered NSCC Ca^2+^ influx.

Interestingly, the amplitude of the phasic contraction (performed in 0 mM Ca^2+^ conditions) was not different between SERCA2^a/b^ and SERCA2^b/b^ segments. However, the area under the curve (AUC) was significantly lower in SERCA2^b/b^ segments. This was mainly due to the faster decay of force in SERCA2^*b*/b^ segments, which is not due to re-uptake of contractile Ca^2+^ to the SR via SERCA, but rather to the extrusion of intracellular Ca^2+^ via the plasmalemmal Ca^2+^ pump (PMCA) (Leloup et al., 2015). Furthermore, the spontaneous leakage of contractile Ca^2+^ from the SR assessed by PE phasic contractions upon various duration of 0 mM Ca^2+^ challenges consistently showed that the AUC of the phasic contraction was smaller in SERCA2^b/b^ mice suggestive for a lower contractile Ca^2+^ content of the SR stores. Additionally, segments of SERCA2^b/b^ mice spontaneously lose their contractile Ca^2+^ slower in function of the duration of the 0 mM Ca^2+^ challenge, suggesting an altered ratio of Ca^2+^ release and removal (Michiels et al., 2015).

Conversely, the tonic contraction induced by the re-addition of external Ca^2+^ tended to be larger in SERCA2^b/b^ segments, especially after addition of diltiazem that blocks VGCC. This again suggests that the difference between both strains relates to NSCC Ca^2+^ influx upon store emptying. When Ca^2+^ was added again to the organ bath, the SR Ca^2+^ stores refilled mainly through Ca^2+^ influx via VGCC and subsequent re-uptake of Ca^2+^ in the SR Ca^2+^ stores via SERCA (Leloup et al., 2015). The refilling of the SR store was less in aortic segments of SERCA2^b/b^ mice, which is in line with the reported twofold lower catalytic turnover rate of SERCA2b compared to SERCA2a and the lower expression of total SERCA protein in SERCA2^b/b^ aortic segments (Ver Heyen et al., 2001; McIvor et al., 2018). As mentioned before, L-type (VGCC) Ca^2+^ channels play a prominent role in refilling of the SR Ca^2+^ stores (Leloup et al., 2015), hence, when 35 μM diltiazem was added during the refilling period, phasic contractions by PE were significantly reduced. Moreover, this reduction was most pronounced in SERCA2^a/b^ segments, thereby confirming the putative role of VGCC in SR refilling.

We previously reported that the SERCA inhibitor CPA (10 μM) causes large intracellular Ca^2+^ transients in SMC (i.e., in EC-denuded segments or segments treated with the eNOS inhibitor L-NAME), which were accompanied by relative minor contractions (Fransen et al., 2015). In the current study, CPA caused similar effects in aortic segments of SERCA2^a/b^ mice. In SERCA2^b/b^ mice, however, CPA caused significantly larger contraction while the intracellular Ca^2+^ response was of similar magnitude as in SERCA2^a/b^ mice. In other words, in the aorta of SERCA2^b/b^ mice there is a more efficient coupling of CPA-elicited intracellular Ca^2+^ to contraction, which persisted after inhibition of L-type Ca^2+^ channels with 35 μM diltiazem. This observation suggests that intracellular Ca^2+^ responses induced by CPA are differently coupled to contraction in SERCA2^a/b^ and SERCA2^b/b^ aortic segments respectively. Functionally segregated SR Ca^2+^ stores have been described in pulmonary arterial SMC (Clark et al., 2010). In these cells SERCA2b was located predominantly proximal to the plasma membrane while SERCA2a was located centrally. Remarkably, in these cells, the proximal SERCA (SERCA2b) was sensitive to the SERCA inhibitor CPA, whereas the central SERCA (SERCA2a) was CPA-insensitive, but thapsigargin-sensitive. The different localization of both SERCA2a/b Ca^2+^ stores may explain the difference in contractile response between both mouse strains. Moreover, we have previously reported that inhibition of SR Ca^2+^ stores with 10 μM CPA turns a PE-mediated contraction from mainly VGCC-mediated to mainly NSCC-mediated (Fransen et al., 2015). Our data support the observation that inhibition of SERCA2b with CPA results in stronger coupling of NSCC Ca^2+^ influx to contractile responses.

Relaxation of PE-induced contractions by increasing intracellular Ca^2+^ in the EC as with CPA and ACh, were similar in SERCA2^a/b^ and SERCA2^b/b^ mice, suggesting equal activation of eNOS and comparable NO release in both mouse strains. On the other hand, results with ATP that mainly activates purinergic (P2Y) receptors (Guns et al., 2005) suggest that in some conditions EC from SERCA2^b/b^ produce higher amounts of NO or that the SMC of the SERCA2^b/b^ mice, which only possess SERCA2b, are more sensitive to NO. Although EC_50_ values of the exogenous NO donors, DEANO and nitrite, were not significantly different between both mouse strains, there was a tendency for a higher sensitivity of the SERCA2^b/b^ SMC to NO. Since cGMP relaxation is the same in SERCA2^a/b^ and SERCA2^b/b^ mice, this indicates that the direct effect of NO on Ca^2+^ re-uptake (Cohen et al., 1999; Van Hove et al., 2009) via SERCA2a and SERCA2b SR Ca^2+^ stores is different.

Finally, basal NO release, i.e., the difference between the amplitude of PE contractions in the absence and presence of the eNOS blocker L-NAME (van Langen et al., 2012), was attenuated in SERCA2^b/b^ mice. Moreover, the fact that in the presence of levcromakalim, basal NO did not differ between SERCA2^a/b^ and SERCA2^b/b^ mice suggests that the difference in basal NO results in a differential regulation of the VGCC Ca^2+^ influx (Van Hove et al., 2009). Previously, studies with SERCA3 gene-ablated mouse have demonstrated that SERCA3 plays a critical role in EC Ca^2+^ signaling involved in NO release and NO-mediated relaxation of vascular SMC, but not in SMC, consistent with its known distribution (Liu et al., 1997; Paul, 1998). The lack of compensation by SERCA2b for the SERCA3 dysfunction further suggests that they are involved in restoring original Ca^2+^ to specific subcellular compartments (Hadri et al., 2006) and that they serve functionally distinct Ca^2+^ pools in EC. In salivary epithelial cells, SERCA3 and SERCA2b have indeed separate intracellular locations (Lee et al., 1997). Furthermore the apparent K_m_ of SERCA3 for Ca^2+^ is much higher than that of SERCA2b, indicating that SERCA3 might function at higher Ca^2+^ concentrations than SERCA2b (Lytton et al., 1992; Arredouani et al., 2002; Hadri et al., 2006).

## Conclusion

Despite the low incidence of SERCA2a in wild type (SERCA2^a/b^) mice, prevention of the alternative splicing underlying the expression of SERCA2a, resulted in a distinct cardiovascular functional phenotype, i.e., higher PE-induced contractions and increased NSCC Ca^2+^ influx, slower re-filling of the SR Ca^2+^ stores and stronger coupling of NSCC Ca^2+^ influx with contraction, lower basal NO efficacy and slightly increased EC-dependent relaxation. Whether the altered vascular reactivity directly relates to SERCA2a depletion or to indirect compensatory mechanisms will require further investigation. Although in a heterozygous SERCA2 knock-out mouse (SERCA2 ±, 50% reduction of SERCA2), aortic contractility to PE or depolarization (KCl) was not changed (Ishida and Paul, 2005), it was observed that the combination of SERCA2a absence and SERCA2b bisection in the SERCA2b/b mouse model caused an altered vascular (aortic) functional phenotype. At least, the attenuated SERCA2b protein expression in the SERCA2^b/b^ mouse model as compared to “wild-type” SERCA2^a/b^ mice is not sufficient to explain the altered cardiovascular functional phenotype. Overall, given the promise of SERCA2a as therapeutic target in cardiac disease, it is important to carefully consider its role in the vascular system.

## Data Availability Statement

The datasets generated for this study are available on request to the corresponding author.

## Ethics Statement

The animal study was reviewed and approved by the Ethical Committee for Animal Testing (ECD), University of Antwerp. Written informed consent was obtained from the owners for the participation of their animals in this study.

## Author Contributions

PF performed the vascular reactivity experiments, P-JG performed the PCR experiments, JC contributed to the Western blots, and PV provided the mouse model and antibodies for IHC and Western blot. P-JG and PF have jointly written the manuscript, whereas JC and PV contributed to the revision of the text. All authors have read and approved the final version of the manuscript.

## Conflict of Interest

The authors declare that the research was conducted in the absence of any commercial or financial relationships that could be construed as a potential conflict of interest.
