# Supplementary material for: Contractile Behavior of Mouse Aorta Depends on SERCA2 Isoform Distribution: Effects of Replacing SERCA2a by SERCA2b
Source: Front Physiol. 2020 Mar 31;11:282. doi: 10.3389/fphys.2020.00282 (PMC7136392; doi:10.3389/fphys.2020.00282)
Supplement: Supplementary file 1 [file Data_Sheet_1.pdf]

Supplementary Figure

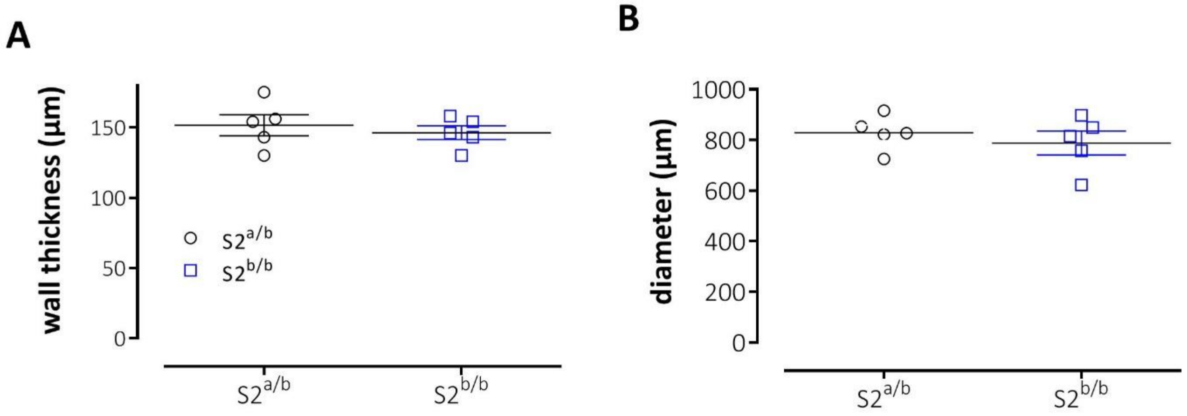

<u>Figure S1</u>. Wall thickness (A) and diameter (B) of aortic segments of SERCA2$^{a/b}$ (S2$^{a/b}$) and SERCA2$^{b/b}$ (S2$^{b/b}$) were similar.

Wall thickness and aortic segment diameter (n=5) were not different between SERCA2a/b and SERCA2b/b mice, suggesting that passive properties of the aortic segments were not changed by replacement of SERCA2a with SERCA2b.
